# An Ishmaelite

**Published:** 1888-04-21

**Authors:** Leslie Keith

**Affiliations:** Author of "The Chilcotes," "Uncle Bob's Niece," "East and West," etc.


### An Ishmaelite.

By Leslie Keith,

Author of "The Chilcotes," "Uncle Bob's Niece," "East and West," etc.

### Chapter III.—The Sword Descends.

To many passers by—to the daily governess going to her task up the next set of steps, for instance, or to the unemployed singing a discordant music in the middle of the way—the stately house in Portland Place might well have seemed the dwelling-place of happiness. It had a look of great prosperity and comfort; beautiful flowers bloomed in all the windows, the furniture was solid and splendid; fine carriages drew up at its door every afternoon and visitors were let in by a punctual footman; a great many entertainments were given in it—dinners that could scarce be matched in London for the costliness and rarity of the viands. But in spite of it all there were far poorer homes that were infinitely better off. The guests who were bidden felt that something was lacking to the feast. The hostess sat with a strained, frightened look on her gentle face, as if she were expecting evil tidings. The host could not always banish the frown that sat now so frequently on his brow. Husband and wife were not at accord, and in the rare evenings when they were alone they avoided exchanging glances, and sat in a chilly silence, too hopelessly estranged even for mutual recrimination.

Helen's fears made her dumb, but it was disappointment that had soured her husband. There were only three inmates now on the upper floors. The nurseries had long since been disused and Miss Plumtree had gone, though her reign extended till the hour of Nellie's emancipation from the schoolroom. Arthur was at Harrow, and was to go thence to Oxford, where Fred already was. But though the household had diminished, a lodger whom nobody had invited had taken up his quarters; care was there, grim care, sitting with the master in the library and with the mistress in the drawing-room; it was only Nellie, pretty, slim, dark-eyed Nellie who was happy, tasting the delights of her first season.

Care took the shape of Fred in the father's heart and the mother's. It was their strife over him that divided them. The father, severe over his lapses, repressed and reproved the boy, and the mother pitied and petted him in secret. Fred's record had not been satisfactory. He came out of the hands of the disciplinarian to whom Cousin Fanny had condemned him rather sullen, and with developed ideas as to the means by which the disagreeable consequences of taking one's own pleasure might be evaded. Mr. Jennings did not easily succeed in running Fred into that mould which he kept for refractory youth. Fred declined to become a model young gentleman on the Jennings pattern, and the reports which the master sent home were tinctured with the bitterness of his failure. Fred had good abilities, but he would not apply; and Mr. Jennings regretted to perceive in him a want of openness and perfect candour, which neither kindness nor severity had any power to correct.

The same tidings coming again and again embittered the father, and his coldness and sternness shut the lad's heart finally against him. Perhaps even then it was not too late for the boy's affection to be won, if it had been sought in the right way. Fred was naturally loving, as many weak people are, with an instinctive desire to cling. To his mother and cousin he showed his best side, and it was a very winning side. He was a handsome young fellow, tall and too slender as yet, but with promise of breadth; his mouth had gained some of the firmness it had lacked in childhood, and his eyes were dark. They could be very tender when they rested on his mother; he loved her passionately, and turned to her in all his troubles. Helen secretly sent him money, and helped him when he got into scrapes, and loved him always fondly but unwisely, shielding him when a braver woman would have let him learn by suffering.

When Mr. Eastgate found out to what quarter his wife's handsome allowance was diverted there was a terrible scene. He poured his scorn and fury, and the bitterness of his own aching heart out on her. "Did she want to ruin her own son? to be in debt, and in debt for such a cause was an indelible disgrace!" Fred's difficulty certainly had an ugly look. Helen had shrunk from hearing of it, though she had gone to the rescue; but her husband's rage scorched her. She started from him with a sickening sense of repulsion and defeat. If he had beaten her she could have borne it better.

"I must request in future that your dressmaker's and milliner's bills are sent to me for payment," he said. "You can order what you will, but your allowance and Nellie's also will be stopped till you know how to spend money without adding by it to my shame."

It was a hard thing even for a hard man to do. It wounded his pride; but his need of conquest demanded it. In a distant sort of way he was sorry for his wife's humiliation, but he was sorrier for his own in having a wife who thwarted him and undermined his schemes, and something of the contempt and gall with which he regarded his son began to creep into his thoughts about Helen.

"If I had married some strong country girl in my own degree," he began to say to himself. When "if" creeps in, love's reign is over.

About this time the venerable Lady Blackrock died. She sent for her granddaughter, Mrs. Eastgate, at the last. The indomitable old lady was sitting up in bed, with a large frilled nightcap round her keen, shrivelled old face. She sent Death about his business while she uttered her last commands.

"I have sent for you, Helen," she said, "to give you my blessing. You have done your duty, and that will be a comfort to you when you are dying like me. You got over that foolish fancy for Jack Fairfield, who hadn't sixpence beyond his pay, and you took Joseph Eastgate, who, considering his origin, has behaved very well indeed; and you have married your sister Arabella most respectably. I am pleased with you, my dear. You are a rich woman, and I am not going to leave you any of my poor little savings; but I think you will appreciate your old grandmother's approval."

Helen put her head down on the pillow; she said faintly through her tears that she was glad to have pleased her grandmother, but she felt no reviving glow of comfort in her own heart. She had deliberately bartered her happiness and there was no compensation for her.

"Done my duty," she said sadly to herself. "I have spoiled two lives; three perhaps." (Jack Fairfield had never married.) "I have been wicked, not good."

At Oxford Fred's allowance was carefully calculated, and limited to his reasonable needs; and with no hedge but this to keep him from temptation, he was launched on that world of freedom to rise or to sink.

"Going to breed the boy to your own business?" asked Mr. Mack, who had once upon a time been a power in the city too.

"I don't know." Mr. Eastgate tried to hide his disappointment under a show of indifference. "He doesn't seem to have any fancy for it as yet. But he shan't say that I haven't given him every advantage. Arthur, my brother Lawrence's boy, has more aptitude for business."

"Well, there's time enough for him to choose, if you're set on giving him a college career; though you and I got on well enough without it, and were put in harness a good deal earlier."

"Ah, times are changed since then. We were made of tougher stuff."

It was scarcely an additional shock to the father when Fred refused to enter the City firm. His loss of faith in his son was too complete for him to feel anything

Army Medical Library but a quickening of contempt over this resolution. "He despises the business I made with my own hands; it is my punishment for marrying into a class that looks down on trade while its does not scruple to share its profits."

It was not that Fred despised the business, but that he had a inconquerable repugnance to any career that would bring him into daily contact with his father. The hope of reconciliation was at an end by the time Fred went to Oxford. He felt nothing but relief in quitting his father's house, except that he left his mother and Nellie there; he meant to work and win honours for his mother's sake; when he kissed her as he went away, he vowed hotly that he would never shame her; that he would retrieve his character in his father's eyes. But he had not counted on the weakness of moral fibre which made the fight harder for him than for others. Oxford treats her sons as men, with a man's responsibility and freedom of choice, and it turns out a good many failures as well as some shining successes.

Fred felt to the full that fascination and charm in the place which all must feel; liberty was sweet, and he drank it to the dregs; he became a sort of leader, with a faithful band of disciples, but they were not among the reading men. His tastes were not low, but they were tolerably expensive; his dress was the standard of fashion in his set, and his little dinners and wines had a reputation just as his father's solid feasts had at home; his boat was the nattiest on the river, and his etchings and rare editions were the envy of all the undergrads of his acquaintance. All these things meant money, and money which certainly did not come out of his father's pocket. Whence did it come? For a couple of years it was plentiful enough, and then there began to come the inevitable time of straitness—the time of duns, of bills showered down for long-forgotten luxuries, of creditors no longer obsequious, of friends beginning to look askance and to fall away. The Jews would have none of him, even at a modest hundred per cent., his luck at cards had failed him, the dons and the dean looked on him as a black sheep, and many ugly whispers were afloat. It was the old story; the prodigal had gone very gaily to the far country and was come now to the husks. But for this poor prodigal there was no father's house to come back to.

One day, in the early spring of the year, Mrs. Eastgate and Nellie were suddenly startled by Fred's arrival. Mrs. Eastgate was sitting languidly near the fire—she was more or less of an invalid now—and Nellie had come in from her morning ride in the Row, and still wore her habit. Fred burst in, looking very ill and dreadfully pale and haggard. The two women started up in alarm.

"What is it?" Nellie called out wildly, but his mother could not speak. She sank down again in her chair, unable to stand. Easter was late that year, and the men were not down yet; it must be something dreadful that had brought Fred to town.

"Mother!" he said, trying to control himself; "I must have money. I'm in a devil of a mess; I shall be disgraced, ruined, unless I have it at once. It's—it's a debt of honour," he faltered; "you always helped me,"—he stooped and kissed her white lips wildly—"Mother, you won't desert me now?"

She looked beyond him, and her glance met Nellie's despairingly. The sum he named would at any time have been beyond their immediate command, but now—

Nellie was trembling, but her indignation found voice. "We are not trusted," she said; "we are treated like children—all our bills are paid for us."

"Oh!" cried Fred, with great bitterness, "I suppose he thought you would give it to me! Never mind." He was despairing, but his mother's face frightened him, and he tried to rally. "Arthur will help me. He always stands by a fellow; or there's old Mack, who may stump up."

But Arthur had left Harrow and had gone off to Scotland with a friend, and neither sister nor aunt knew his address; but Nellie, suddenly visited by an inspiration, ran out of the room. She flew upstairs, and tremblingly unlocked her dressing-case. Some of the jewels that had belonged to her mother lay there; she seized the cases and opened them feverishly. She was not entrusted with the diamonds, but she quickly selected all that was valuable—a pearl necklace and one or two bracelets and rings. She did not stop to tie up the cases, but rushed down with them in her hand, and imperiously commanded the astonished footman to call her a cab. She did not remember that she still wore her habit till she stumbled over it as she ran across the pavement, and found how sadly it hampered her steps.

In less than half an hour Nellie came back flushed and excited. She went hastily upstairs. "Fred, Fred," she cried, "this isn't all you want, but it may help."

But the drawing-room was empty, except for Mrs. Eastgate, who lay back half fainting in her chair. "Oh, why did you let him go," said Nellie, breaking down all at once and sobbing helplessly. "There must have been ways—I shall have money when I am twenty-one—there must be ways of advancing it. Oh, why was I such a fool!" It was herself the poor girl reproached, as she sat crushed and overwhelmed, sobbing out her grief.

They both knew instinctively that calamity was about to overtake them, and they waited for it in an agony of suspense, listening with sickening dread for Mr. Eastgate's step; they scarcely dared to look in his face, not knowing what they might read there; but it was only gloomy and austere, as it always was now. Nellie sent the money to Oxford with a little letter written in a shaky hand imploring Fred to write, and promising him all he asked, but he made no sign. Two days of awful suspense followed, and then the sword descended.

Nellie had scarcely left her aunt's side in those days; but once, when she had with difficulty persuaded Mrs. Eastgate to go down to lunch, something detained her for a moment upstairs. She was about to follow, when her ear—alert now for any sound—heard the turning of a key in the latch. She listened with a wildly-beating heart. Could it be Fred? The next moment she recognized her uncle's creaking, heavy step.

"It has come," she said to herself. She was so sick that she could scarcely crawl downstairs, and had to support herself by the railing. When she reached the dining-room shesaw her uncle framed there; his profile was towards her, but she noticed the awful livid passion of his face. She caught but two or three words, but they told her everything.

"Your son—a forger and a felon!" Even in that dreadful moment she noticed the cruel stress on the words "*your* son." They had scarcely more than a mechanical sound for her ear as yet—thos other awful words. She had just strength enough to know that she must go to her aunt, and she stole past her uncle like a white, trembling ghost. Mrs. Eastgate lay on the floor in a faint.

(*To be continued.*)

